# Reduced Formation of Oxidative Stress Biomarkers and Migration of Mononuclear Phagocytes in the Cochleae of Chinchilla after Antioxidant Treatment in Acute Acoustic Trauma

**DOI:** 10.1155/2011/612690

**Published:** 2011-09-25

**Authors:** Xiaoping Du, Chul-Hee Choi, Kejian Chen, Weihua Cheng, Robert A. Floyd, Richard D. Kopke

**Affiliations:** ^1^Hough Ear Institute, Oklahoma City, OK 73112, USA; ^2^Audiology and Speech-Language Pathology, College of Medical Sciences, Catholic University of Daegu, Kyungsansi, Republic of Korea; ^3^Spatial Orientation Center, Naval Medical Center, San Diego, CA, USA; ^4^Experimental Therapeutics Research Program, Oklahoma Medical Research Foundation, Oklahoma City, OK 73104, USA; ^5^Department of Otolaryngology, University of Oklahoma Health Sciences Center, Oklahoma City, OK 72104, USA

## Abstract

*Objective*. Inhibition of inflammation and free radical formation in the cochlea may be involved in antioxidant treatment in acute acoustic trauma. *Procedure*. Chinchilla were exposed to 105 dB sound pressure level octave band noise for 6 hours. One group of chinchilla was treated with antioxidants after noise exposure. Auditory brainstem responses, outer hair cell counts, and immunohistochemical analyses of biomarkers in the cochlea were conducted. *Results*. The antioxidant treatment significantly reduced hearing threshold shifts, outer hair cell loss, numbers of CD45^+^ cells, as well as 4-hydroxy-2-nonenal and nitrotyrosine formation in the cochlea. *Conclusion*. Antioxidant treatment may provide protection to sensory cells by inhibiting formation of reactive oxygen and nitrogen products and migration of mononuclear phagocytes in the cochlea. The present study provides further evidence of effectiveness of antioxidant treatment in reducing permanent hearing loss.

## 1. Introduction

Metabolic oxidative stress plays a significant role in acute acoustic trauma (AAT) and provokes the production of reactive oxygen species (ROS) and reactive nitrogen species (RNS) in the inner ear [1–4]. In the cochlea, free radicals and their products emerge or increase immediately after high level noise exposure as well as a second peak that occurs 7–12 days [2–8]. The direct relationship between the formation of free radicals and AAT is not fully understood. Free radicals may directly cause cochlear dysfunction and DNA damage [5, 9, 10] and induce cell death in the inner ear [11–14]. More importantly, a self-perpetuating reaction of free radicals and ROS on lipid molecules may be responsible for continuing cell damage after noise exposure [15]. ROS and RNS may also cause mitochondrial membrane injury, cytochrome C release, and ischemia/reperfusion damage and trigger apoptotic cell death in the inner ear [15–19].

Based on these studies, a number of antioxidants were studied either to enhance intrinsic cochlear stress defenses or as exogenous antioxidants and have been successfully used to prevent AAT through systemic or local application in several animal models [20–27]. More importantly, postexposure treatment in animal models [21, 24, 27, 28] provides a new possible use of the antioxidants to treat AAT in the clinic in the future. 

Antioxidants target different pathways in the inner ear. For example, *N*-acetyl-L-cysteine (NAC) provides cysteine for synthesis of reduced glutathione (GSH), which is an important antioxidant compound in mitochondria [24, 28–30]. Acetyl-L-carnitine (ALCAR) can restore mitochondrial membrane integrity and reduce ROS production [16, 25, 29]. Protective mechanisms of 4-hydroxy phenyl *N*-*tert*-butylnitrone (4-OHPBN), a major metabolite of phenyl *N*-*tert*-butylnitrone (PBN), are still unclear although it has been successfully used in the treatment of AAT [21, 31]. PBN is a spin trapping agent, and its phenyl ring reacts with hydroxyl radicals [32]. Biological functions of PBN may involve the reduction of oxidative stress and inflammation, as well as attenuation of glutamate excitotoxicity [33, 34]. Although PBN was unable to reduce the auditory threshold shifts induced by noise alone in rats [35, 36], the derivative, 4-OHPBN, alone or in combination with other antioxidants can effectively treat AAT in chinchilla, suggesting 4-OHPBN may have different biological functions compared to PBN [21]. A three-drug combination (NAC + ALCAR + 4-OHPBN) was significantly better than any single antioxidant treatment as reflected by hearing testing through auditory brainstem responses (ABRs) and outer hair cell (OHC) counts [21]. However, the underlying cellular and molecular mechanisms of these antioxidants in treating AAT have not yet been established. 

At least 2 free radical products have been used as biomarkers of oxidative stress to label ROS and RNS activity in the inner ear and to evaluate effects of antioxidant treatment after noise exposure [6, 8, 27, 37]. Four-hydroxy-2-nonenal (4-HNE) is an indicator of oxidative damage formed as an abundant product of polyunsaturated fatty acid oxidation and decomposition. 4-HNE reacts extensively with DNA and proteins, depletes intracellular GSH, and alters many cell signaling cascades [38]. Delayed 4-HNE formation has been found in the organ of Corti of guinea pigs and was shown to peak 7–10 days after a noise exposure of 120 dB sound pressure level (SPL) for 5 hours [8]. Nitrotyrosine (NT), a marker of nitric oxide (NO) production, is formed by nitration of a tyrosine residue in proteins [39]. Another free radical biomarker, malondialdehyde, was found in cochlea immediately after noise exposure as well as a second peak at 12 days [6, 7, 37]. We have examined these three free radical biomarkers in the present study. Other biomarkers that were also examined include cytoplasmic cytochrome C [40, 41], inducible nitric oxide synthase (iNOS) [42, 43], and caspase 3 [17, 40].

In recent years, the relationship between inflammation and oxidative stress has been extensively studied in various organs and systems, that is, pulmonary and cardiovascular systems, CNS, liver, and muscle [44]. In the inner ear, inflammatory cells have been found in different structures of the cochlea after noise exposure [45–47]. For example, dendritic macrophages were noted in the organ of Corti 5 days after noise exposure [45]. A large increase in the number of CD45^+^ cells was found in the spiral ligament and spiral limbus 1–14 days after noise exposure [46, 47]. This inflammatory response may be involved in propagating cellular damage in the cochlea after noise exposure [46]. Because oxidative stress and inflammation are traditionally associated with AAT, we used an anti-CD45 antibody as an inflammatory marker to examine the effects of antioxidant treatment on inflammation in the cochlea. 

In the present study, the cochleae were examined using semiquantitative immunohistochemical analyses 10 days after noise exposure, a second time point that free radicals reached a maximum [8], to evaluate the effectiveness of antioxidant treatment in AAT using inflammatory, oxidative stress, and cell death biomarkers.

## 2. Materials and Methods

### 2.1. Animals

The experimental procedures were approved by the Institutional Animal Care and Use Committees of the Office of Naval Research and the Oklahoma Medical Research Foundation (OMRF). Eighteen female adult chinchilla laniger weighing 500–850 g (3 to 5 years old) were purchased from Moulton Chinchilla Ranch (Rochester, MN) and housed in the OMRF animal facility with free access to a standard chinchilla diet (Mazuri Chinchilla Diet, 5MO1, PM1 Nutrition International Inc., Brentwood, MO) and tap water throughout the experimental periods. The ambient noise level in the animal facility was 54.7 dB (A)/20.0 uPa. The chinchilla were randomized into 3 groups (*n* = 6 in each group): animals in the noise exposure plus carrier solution (dimethyl sulfoxide, polyethylene glycol 400, and saline) and noise plus treatment (noise/treatment) groups were exposed to a 105 dB SPL octave-band noise for 6 hours (detailed below). Animals in the normal control group were not exposed to noise.

### 2.2. Noise Exposure

For noise exposure, two animals at a time were placed in two small wire restraint cages on a wooden plate. They were exposed to a 105 dB SPL octave-band noise centered at 4 kHz for 6 h in a sound isolation booth (Industrial Acoustics Company, New York, NY). The noise generation was detailed in our previous report [21]. Briefly, the noise was generated, filtered by a Tucker Davis Technologies (TDT, Alachua, FL) device, amplified (QSC audio PLX 3402 power amplifier, Costa Mesa, CA), and transduced with an acoustic speaker (JBL 2350, Northridge, CA). The dropoff of noise energy outside the octave-band noise being produced was 20–25 dB/octave. The speaker was suspended from the ceiling of the sound booth and positioned directly above the wire cages. A condenser microphone (B&K 2804, Norcross, GA) coupled to a preamplifier and the PULSE software system (B&K Sound & Vibration Measurement) was placed between the two wire cages at the level of the animals' heads to continually monitor the noise level during noise exposure.

### 2.3. Injection of Antioxidants

Animals in the noise/treatment group received an initial injection 4 hours after the noise exposure and then twice a day for the following 2 days. Animals in this group received 20 mg/kg of 4-OHPBN dissolved in dimethyl sulfoxide, polyethylene glycol 400, and saline, 50 mg/kg of NAC (Hospira Inc., Lake Forest, IL), and 20 mg/kg of ALCAR (Sigma-Aldrich Inc. St. Louis, MO). These agents were intraperitoneally administered. In the noise exposure and the normal control groups, equal volumes of carrier solution were injected at the same time points as in the noise/treatment group. The three-drug combination was used in the present study since this combination showed the best hearing protection as reported in our previous study [21].

### 2.4. Measurement of Auditory Brainstem Responses

ABR thresholds for both ears of each animal were measured before initial noise exposure (baseline threshold), immediately after, and then 10 days after noise exposure. ABR threshold shift was obtained as the difference between the baseline threshold and the final ABR threshold measured 10 days after noise exposure. The ABR recordings procedure was detailed in our previous report [21]. Briefly, animals were lightly anesthetized with ketamine (20 mg/kg) and xylazine (1 mg/kg). The ABR thresholds were determined by decreasing the sound intensities of the tone pips (5 ms duration and 1 ms Blackman rise/fall ramp at 0.5, 1, 2, 4, 6, and 8 kHz) first at 10 dB steps until near the threshold and then 5 dB ascending steps. Threshold was defined as the midpoint between the lowest level of a clear response and the next level of nonresponse. The investigators obtaining the ABR thresholds were blinded as to the identity of the animal groups.

### 2.5. Tissue Collection and HC Counting

After the last ABR measurement, the chinchilla were euthanized with an overdose of ketamine and xylazine and then intracardially perfused with 0.1 M phosphate buffered saline (PBS, pH 7.2), followed by 4% paraformaldehyde in PBS. Cochleae were removed and postfixed in the same fixative overnight and then washed in PBS and stored in the buffer at 4°C. The right cochlea from each animal was used for whole mount and TRITC-phalloidin staining for HC counting. Percentages of HCs were obtained by dividing the OHC count from the experimental animals by the HC count from normal control animals for each cochlear section [48]. Finally, the percentage of missing HCs was plotted as a function of percent distance from the cochlear apex by entering inputs into a worksheet to construct a cytocochleogram [21, 48]. An equation of cochlear frequency-place map (*F* = 125*e* ^0.051*d*^, where *F* is the frequency in Hz and *d* is percent distance from the apex) was used to evaluate HC losses at specific frequencies of 2, 4, 6, and 8 kHz [21, 49, 50]. The left cochlea from each animal was processed for immunohistochemical analysis (detailed below).

### 2.6. Immunohistochemical Analysis

Cochleae were washed in dH_2_O three times and immersed in 10% EDTA for 2 weeks with 3-4 solution changes. After decalcification, the cochleae were cryoprotected in 30% sucrose in PBS at 4°C overnight, embedded in Tissue-Tek (Sakura Finetek USA Inc. Torrance, CA) and serially sectioned in a perimodiolar plane with a Thermo Cryotome (Thermo Fisher Scientific, Inc. Waltham, MA) at 18–20 *μ*m. Serial sections were mounted onto gelatin precoated slides.

For fluorescence immunohistochemical staining, the sections were washed 3 times with PBS, blocked in 1% bovine serum albumin (fraction V) and 1% normal goat serum in PBS for 1 hour, and permeabilized in 0.2% triton X-100 in PBS (PBS/T) for 30 minutes. The sections were then incubated with a primary antibody (1:200 mouse anti-nitrotyrosine IgG, Upstate, Lake Placid, NY; 1 : 100 rabbit anti-4-hydroxy-2-nonenal Michael adducts IgG, chemically reduced, EMD Chemicals, Inc. Gibbstown, NJ; 1 : 500 rabbit anti-malondialdehyde polyclonal IgG, Chemicon International, Inc. Temecula, CA) for 2 hours. After washing with PBS/T, Alexa Fluor 594 donkey antimouse or antirabbit IgG (1 : 1000, Invitrogen, Carlsbad, CA) was applied onto the slides for 1 hour. After rinsing with PBS, 4′, 6-diamidino-2-phenylindole (DAPI, 1 : 20,000) was used for nuclear staining. A coverslip was applied with ProLong Gold Antifade Reagent (Invitrogen, Carlsbad, CA). To eliminate possible artificial effects on fluoresce intensity that may be caused by different staining conditions, each run of immunostaining included the same number of slides from each group. Images were collected with fluorescence microscopy (Olympus BX51, Melville, NY) or confocal microscopy (Leica SP2 Confocal Microscope, Heidenberg, Germany). Other primary antibodies used in the fluorescence immunohistochemical staining include rabbit anticaspase 3 IgG (1 : 50, Millipore, Temecula, CA), mouse anti-iNOS IgG (1 : 100, Abcam Inc, Cambridge, MA), and rabbit anticytochrome C (1 : 50, Cell Signaling Technology, Danvers, MA).

To study mononuclear phagocyte migration in the cochleae, mouse anti-CD45 IgG1 (1 : 25, BD Pharmingen, San Jose, CA) was incubated with cochlear sections overnight. After PBS/T washing, biotinylated antimouse IgG (1 : 200, Vector Laboratories, Inc. Burlingame, CA) was applied onto the slides for 1 hour, and Vectastain ABC and DAB kits (Vector Laboratories, Inc. Burlingame, CA) were then used for the immunolabeling visualization. Immunopositive cells had a brown reaction product. Images were collected by light microscopy (Olympus BX51, Melville, NY).

A negative control was obtained by omitting the primary antibody in both fluorescence and ABC immunohistochemical analyses.

### 2.7. Quantitation of Immunostaining and Statistical Analysis

A modified semiquantitative procedure [51] was employed to quantify CD45^+^ cells in the cochleae. Images were taken by light microscopy (40x objective) from cross-sections of the stria vascularis in every other section, and the distance between two images was about 400 *μ*m to ensure nonduplicate counting. Five to six images were collected from each cochlear turn. CD45^+^ immunostaining density was obtained by dividing the number of positive cells in each cross-section of the stria vascularis by the cross-sectional area of the stria vascularis (CD45^+^ density = number of CD45^+^ cells/size of stria vascularis (per mm^2^)). The sizes of the cross-sections of the stria vascularis were measured by drawing a line along the border of the stria vascularis with ImageJ software (National Institutes of Health). Only big (>5-6 *μ*m) dark brown dots were counted to avoid counting melanin in the stria vascularis.

Measurement of 4-HNE relative fluorescence intensity in the organ of Corti was conducted with LAS AF Lite software (Leica Microsystems CMS GmbH, Heidenberg, Germany). Two to three images were collected from each turn from midmodiolar sections of each cochlea by fluorescence microscopy using the same camera settings. The images were taken only from midmodiolar sections so that a similar shape and size of the organ of Corti could be measured in all animals. The distance between two images was about 200–400 *μ*m to ensure non-duplicate measurement. Relative fluorescence intensity was measured with the software by drawing a line along the border of the organ of Corti, from which the mean pixel intensity of the labeling was derived. 

A modified semiquantitative procedure [52] was employed to count an NT immunostaining index in the spiral ligament in three turns. Images were taken from the spiral ligament by fluorescence microscopy (40x). The images were taken from every other section, and distance between two sections was about 400 *μ*m to ensure non-duplicate counting. Five to six images were collected from each turn. The total number of cells (number of nuclei stained by DAPI) and NT positive cells in the image were counted using the ImageJ software. An immunostaining index was obtained by dividing the number of NT positive cells by total number of cells within the image. 

The cell counting and the intensity measurement were conducted by a technician who was blinded as to the identity of the animal groups. ABR and cell counting data are reported as mean ± SEM. One-way ANOVA (SPSS 14.0 for windows) was used to statistically analyze the ABR threshold shift and the missing OHC data between noise exposure and noise/treatment groups and to determine if there were statistically significant differences among three groups in CD45**^+^** immunostaining density, 4-HNE relative fluorescence intensity, and NT immunostaining index. When a significant difference was found in ANOVA, a post hoc test (Tukey HSD) or a paired sample student *t*-test was used for mean comparisons between groups. A *P* value of less than 0.05 was considered to be significant in the statistical analyses.

## 3. Results

### 3.1. Antioxidant Treatment Attenuated Hearing Threshold Shifts

**Figure 1:**
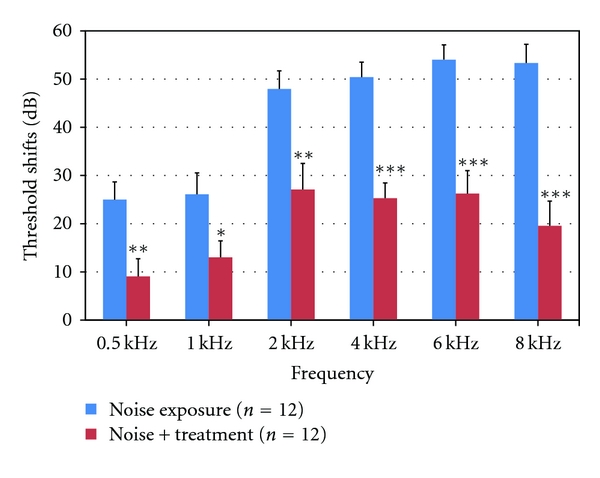
Average ABR threshold shifts at each frequency (0.5–8 kHz) 10 days after noise exposure. Hearing threshold shifts were found in both the noise exposure and the noise/treatment groups at all frequencies with greater shifts in the high frequencies (2–8 kHz). Compared to the noise exposure group, significant reductions in threshold shifts were found in the noise/treatment group at all frequencies. There are significant differences in the threshold shifts between the noise exposure and the noise/treatment groups at all frequencies, especially at high frequencies (***, **, and * indicate *P* < 0.001, <0.01, and <0.05, resp. Error bars represent standard error of the means. Number of ears = 12 in each group).

The ABR thresholds were equivalent among three groups prior to noise exposure (*P* > 0.05 for all frequencies, data not shown). Compared to baseline ABR thresholds measured before the noise exposure, there are significant hearing threshold shifts in both the noise exposure and the noise/treatment groups at all frequencies, but with larger threshold shifts found in the noise exposure group, especially at the higher frequencies (2–8 kHz). As shown in Figure 1, the mean ABR threshold shifts in the noise exposure group ranged from ~25 dB at low frequencies (0.5–1 kHz) to ~51 dB at high frequencies (2–8 kHz). In the noise/treatment group, mean threshold shifts ranged from ~10 dB at the low frequencies to ~25 dB at the high frequencies. Compared to the noise exposure group, significant reductions in threshold shifts were found in the noise/treatment group at all frequencies (0.5–8 kHz, *P* < 0.05, <0.01 or <0.001) with greater reductions at the high frequencies (2–8 kHz, *P* < 0.001, Figure 1). The hearing threshold shift average at higher frequencies was ~24 dB in the noise/treatment group and ~51 dB in the noise exposure group (*P* < 0.001). The ABR thresholds were equivalent between two measurements (baseline and thresholds measured before subjects were euthanized) in the normal non-noise exposed control group (*P* > 0.05 for all frequencies, data not shown). These results indicate that antioxidant treatment used in the present study can significantly attenuate hearing loss induced by AAT.

### 3.2. Antioxidant Treatment Reduced OHC Loss

**Figure 2:**
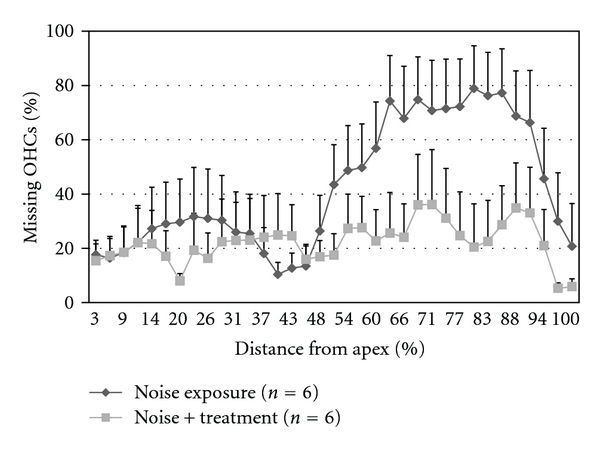
A cytocochleogram representing mean percentage of missing OHCs at the measured percent distance from the cochlear apex in the noise exposure and the noise/treatment groups. Reduced OHC loss was found in the noise/treatment group compared to the noise exposure group, especially at the 55–100% distance from cochlear apex. There is a significant difference between these two groups in average OHC loss in the cochleae (*P* < 0.001, error bars represent standard error of the means. Number of cochleae = 6 in each group).

As shown in the cytocochleogram in Figure 2, the majority of missing OHCs in the noise exposure group were located at the region of 55–95% of the distance from cochlear apex, representing frequencies ranging from 2 to12 kHz. This may mirror the HC damage/death pattern in the cochlea exposed to a narrow band noise, in which HC death had spread apically and basally from the initial regions of injury [53]. Reduced OHC loss was found in the noise/treatment group compared to the noise exposure group, especially at the 55–100% distance from cochlear apex. There was a significant difference between these two groups in the mean percentages of OHC loss in the cochleae (*P* < 0.001). Average percentages of missing OHCs at regions corresponding to cochlear frequencies ranging from 2 to 8 kHz were ~60% in the noise exposure group and ~25% in the noise/treatment group. There was a significant difference between these two groups in the average OHC loss at the high frequency region (*P* < 0.001). These results indicate that antioxidant treatment significantly reduced OHC loss from AAT.

### 3.3. Antioxidant Treatment Reduced CD45^+^  Cell Migration into the Lateral Wall

**Figure 3:**
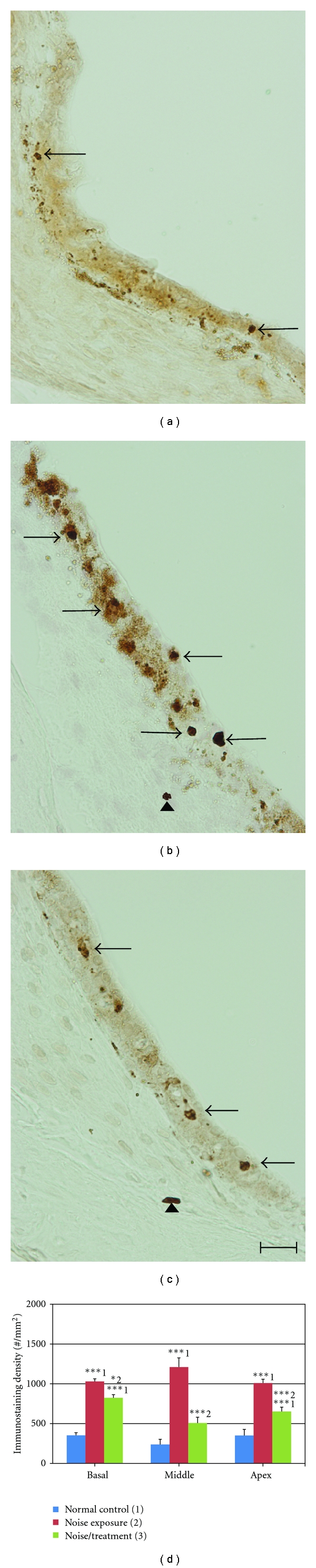
CD45^+^ immunostaining and immunostaining density in the stria vascularis. Examples of CD45^+^ immunostaining images obtained from the stria vascularis in the middle turn of cochleae from the normal control (a), noise exposure (b), and noise/treatment (c) groups by light microscopy. Few CD45^+^ cells were found in the stria vascularis of the normal group (arrows in (a)). There was a large increase in the number of CD45^+^ cells in the stria vascularis of the noise exposure group (arrows in (b)). The antioxidant treatment significantly decreased the number of CD45^+^ cells in the stria vascularis (arrows in (c)). CD45^+^ cells were also found in the spiral ligament of the noise and the noise/treatment groups (arrowheads in b and c), but not in the normal control group. The results of the immunostaining density measurement are shown in (d) (number of cochleae = 6 in each group). There were significant differences among the three groups and in pairwise comparisons in the three groups in each turn. The numbers (1, 2, 3) in (d) indicate the normal control, noise exposure, and noise/treatment groups, respectively. Scale bar = 20 *μ*m in (c) for (a)–(c) (error bars represent standard error of the means. *, **, *** indicates *P* < 0.05, 0.01, 0.001, resp. Number of cochleae = 6 in each group).

As shown in Figure 3, a few CD45^+^ cells were found in the stria vascularis of the normal control group (arrows in Figure 3(a)), but not in the spiral ligament or in the organ of Corti (data not shown). However, a significantly increased number of CD45^+^ cells were detected in the stria vascularis of the noise exposure group (arrows in Figure 3(b)). CD45^+^ cells in the stria vascularis were primarily found in the basal and intermediate cell layers with a few in the marginal cell layer. Compared to the noise exposure group, a significantly decreased number of CD45^+^ cells were found in the stria vascularis of the noise/treatment group (arrows in Figure 3(c)). A few CD45^+^ cells were also found in the spiral ligament of the noise exposure and the noise/treatment groups (arrowheads in Figures 3(b) and 3(c)). Figure 3(d) displays the results of the CD45^+^ immunostaining density measurement in the stria vascularis at each turn of the cochleae of all three groups. Two sets of ANOVA and post hoc tests (Tukey HSD) were conducted in the statistical analyses. Firstly, we analyzed the CD45^+^ immunostaining densities among the three groups at each turn (basal, middle, or apical). The results indicated that there were statistically significant differences among the three groups at each turn (all *P* < 0.001). The post hoc test (Tukey HSD) demonstrated that there were significant differences in pairwise comparisons among the three groups (normal control versus noise exposure; normal control versus noise/treatment; noise exposure versus noise/treatment) at each turn (*P* < 0.05 or <0.001). Then, we analyzed the CD45^+^ immunostaining densities among the three turns in each group. The levels of CD45^+^ cells at three turns are equivalent in the normal control group (all *P* > 0.05), as well as in the noise exposure group (all *P* > 0.05), but a significantly reduced number of CD45^+^ cells were found in the middle turns compared to those in the basal turn in the noise/treatment group (*P* < 0.01). These results suggest that antioxidant treatment significantly reduced migration of CD45^+^ cells into the stria vascularis of three turns of cochleae and that the treatment was more efficient in the middle turn than in the basal turn. CD45^+^ cells were occasionally seen in the organ of Corti in the noise exposure and noise/treatment groups (data not shown). No CD45^+^cells were found in the spiral ganglia of all three groups (data not shown).

### 3.4. Antioxidant Treatment Reduced 4-HNE Formation in the Organ of Corti

**Figure 4:**
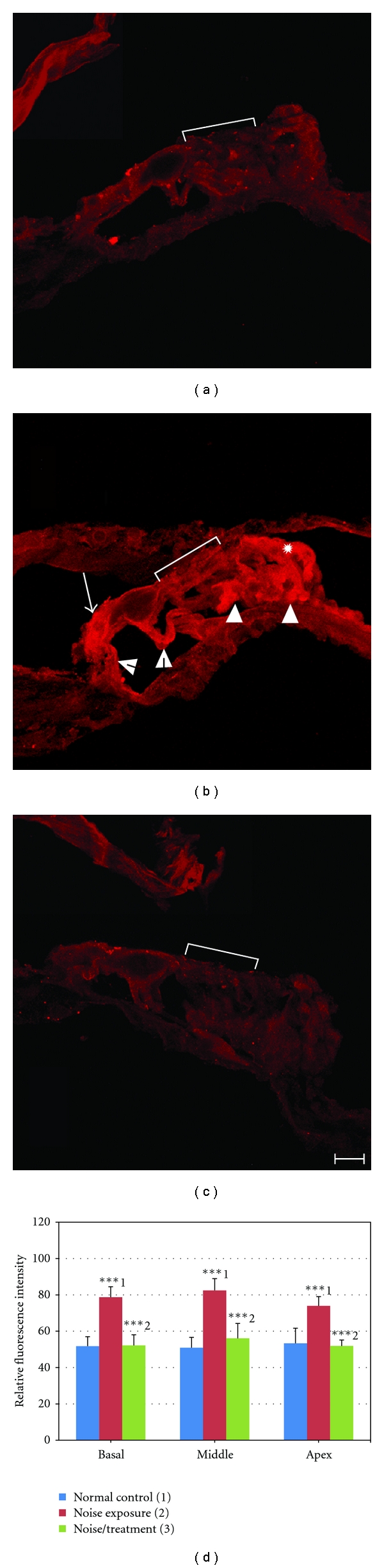
4-HNE immunostaining and relative fluorescence intensity in the organ of Corti. Examples of 4-HNE immunostaining images obtained from the organ of Corti in the middle turn of cochleae from the normal control (a), noise exposure (b) and noise/treatment (c) groups by confocal microscopy using the same microscopic and image collecting settings. No significant 4-HNE staining was found in the organ of Corti of the normal control group (a). Strong positive staining was seen in IHC (arrow in (b)), inner and outer pillar cells (open arrowheads in (b)), Deiters cells (arrowheads), and cells of Hensen (starburst) and Boettcher of the noise exposure group. The OHC region also had positive, but relatively weak 4-HNE immunostaining (bracket in B). Significantly lower HNE staining was found in the organ of Corti in the noise/treatment group (c). The results of the relative fluorescence intensity in the organ of Corti measured by LAS AF Lite software are shown in (d). High fluorescence intensity was found only in the organ of Corti of the noise exposure group. There were significant differences among three groups and between the normal control and the noise exposure groups, as well as between the noise exposure and the noise/treatment groups (***indicates *P* < 0.001). However, there was no significant difference between the normal control and the noise/treatment groups (*P* > 0.05). There was no significant difference among three turns within each group (*P* > 0.05). The numbers (1, 2, 3) in (d) indicate the normal control, noise exposure and noise/treatment groups, respectively. Brackets indicate the OHC region in (a)–(c). Scale bar = 20 *μ*m in (c) for (a)–(c) (Error bars represent standard error of the means. Number of cochleae = 6 in each group).

No positive 4-HNE staining was found in the cochleae of the normal control group (Figure 4(a)). However, in the noise-exposed cochleae, positive staining was found in the organ of Corti in all three turns. In the basal turns, positive 4-HNE staining was shown in inner hair cells (IHCs) as well as Deiters and Hensen cells while most OHCs were missing in this area, which is consistent with the OHC counting data. In the middle turn, strong positive staining was found in IHCs (arrow in Figure 4(b)) and in most supporting cells (SCs, inner and outer pillar cells, Deiters cells, and cells of Hensen and Boetthcher, arrowheads and starburst in Figure 4(b)). The OHC region had relative weak 4-HNE immunostaining (bracket in Figure 4(b)). 4-HNE immunostaining in the organ of Corti of the noise/treatment group was more similar to the staining of the normal control group (Figure 4(c)). There were statistically significant differences among the three groups in the relative fluorescence intensity in each turn (ANOVA, all *P* < 0.001). In each turn, significant differences were found between the normal control and the noise exposure groups, as well as between the noise exposure and the noise/treatment groups (*P* < 0.001), but not between the normal control and the noise/treatment groups (Tukey HSD, *P* > 0.05, Figure 4(d)). There was no significant difference among basal, middle, and apical turns within each group (all *P* > 0.05). These results indicate that antioxidant treatment significantly reduced the formation of 4-HNE to control levels in the organ of Corti at all three turns. No positive 4-HNE immunostaining was found in the lateral wall of cochlea or in the spiral ganglia of all three groups (data not shown).

### 3.5. Antioxidant Treatment Inhibited NT Formation in the Spiral Ligament

**Figure 5:**
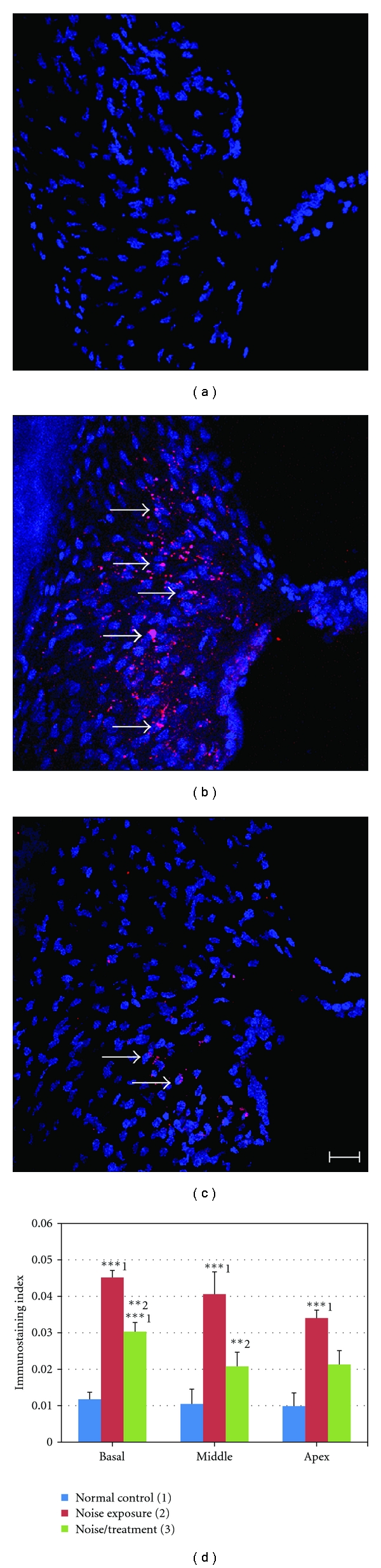
NT immunostaining and immunostaining index in the spiral ligament. Examples of NT immunostaining images obtained from the spiral ligament in the middle turn of cochleae in the normal control (a), the noise exposure (B), and the noise/treatment (c) groups by confocal microscopy. No significant NT immunostaining was found in the spiral ligament of the normal control group (A). A large number of NT positive cells were found in the spiral ligament of the noise exposure group (arrows in (b)) while a significantly decreased number of NT positive cells were found in the noise/treatment group (arrows in (c)). The results of the immunostaining index are shown in (d). A high NT immunostaining index was found in the spiral ligament in all three turns of the noise exposure group. There were significant differences between the normal control and the noise exposure groups in the cochlear three turns, and between the noise exposure and the noise/treatment groups in the basal and middle turns. There was no significant difference between the noise exposure and the noise/treatment groups in the apical turn. The numbers (1, 2, 3) in (d) indicate the normal control, noise exposure, and noise/treatment groups, respectively (*** and ** indicate *P* < 0.001 and <0.01, resp.). Scale bar = 20 *μ*m in (c) for (a)–(c) (error bars represent standard error of the means. Number of cochleae = 6 in each group).

As shown in Figure 5(a), no significant NT immunostaining was found in the spiral ligament of the normal control group. However, strong positive staining was found in the spiral ligament of the noise exposure group. The staining was located in the cytoplasm of fibrocytes (arrows in Figure 5(b)). A significantly reduced number of NT positive cells were found in the spiral ligament of the noise/treatment group (Figure 5(c)). Figure 5(d) displays results of the immunostaining index measurement in the spiral ligament in the cochlear three turns. There were statistically significant differences among the three groups in each turn (ANOVA, all *P* < 0.001). The post hoc test (Tukey HSD) demonstrated that there were significant differences between the normal control and noise exposure groups in all three turns (*P* < 0.001), as well as between the noise exposure and the noise/treatment groups in basal and middle turns (*P* < 0.01 or <0.001), indicating that antioxidant treatment significantly inhibited NT formation in the spiral ligament in the basal and middle turns. There was trend toward reduced NT formation in the apical turn; however, there was no statistically significant difference between the noise exposure and the noise/treatment groups in the apical turns. Additionally, there was no significant difference between the normal control and the noise/treatment groups in the middle turn, indicating that the treatment was more efficient in the middle turn than in the basal turn. The level of NT positive cells was equivalent among different turns in each group (all *P* > 0.05). No significant NT immunostaining was found in the organ of Corti or in the spiral ganglion of all three groups (data not shown).

### 3.6. Some Biomarkers Did Not Show Significant Positive Staining in the Cochleae 10 Days after Noise Exposure

Biomarkers for malondialdehyde, cytochrome C, iNOS, and caspase 3 showed no significant positive staining in the cochleae of all three groups. The lack of significant positive expression of these biomarkers in the cochleae might be associated with the time point examined and noise intensity used in the present study. Most of these biomarkers are present in the cochlea early after noise exposure. For example, a high concentration of malondialdehyde was found in the cochlea immediately after noise exposure with a second peak at 12 days [6, 7, 37]. Apoptotic cell death has been found primarily in the OHCs from a few minutes to 4 days after noise exposure [17, 40]. Upregulation of iNOS was found in the cochlea from immediately to about 1 day after noise exposure [42, 43].

## 4. Discussion

This study demonstrates that noise exposure of 105 dB SPL for 6 hours induced an ABR threshold shift of about 40–45 dB in chinchilla. The same noise exposure resulted in only about a 20 dB threshold shift in the animals that received the three-antioxidant combination treatment. Consistent with the functional protection, the treatment significantly rescued 40–50% of OHCs from AAT. These results are consistent with previous reports using the same noise level and duration [21, 28, 29, 31, 54] although the results in the present study were obtained 10 days, instead of 21 days, after noise exposure. The ABR threshold shifts in the present study were higher than those recorded at 21 days after noise by 12–15 dB, which may represent residual temporary threshold shift. Our semiquantitative data have demonstrated that formation of oxidative stress biomarkers and migration of mononuclear phagocytes in the cochleae were significantly reduced by the antioxidant treatment. There are two significant findings in the present study. First, the present study demonstrates that antioxidant treatment can inhibit an aspect of inflammation in the cochlea, and this inhibition was associated with functional and histological recovery with the antioxidant treatment. Second, the present study has provided further evidence that this three-drug combination treatment protects the cochlea from oxidative injury in AAT.

### 4.1. The Role of Inflammatory Response in the Cochlea after Noise Exposure and Antioxidant Treatment

CD45, a common leukocyte antigen, is present on all bone-marrow-derived white blood cells. Normally, only a few CD45^+^ cells are found in the cochlea, but the number of CD45^+^ cells was significantly increased in the noise-exposed cochleae [46, 47, 55]. Consistent with these reports, increases in CD45^+^ cells were found in the stria vascularis and the spiral ligament of the noise exposed cochleae in the present study. Furthermore, the current study has demonstrated that the antioxidant treatment significantly inhibited the migration of CD45^+^ cells into the cochlea by 20–58%, suggesting that inhibition of inflammation appears to be a new mechanism of antioxidant treatment in AAT. The present study has also suggested as others have noted that inhibition of inflammation may be a strategy to treat AAT [56].

Although the previous and present studies have suggested that therapeutic benefits can be obtained from inhibition of inflammation in AAT [56], the role of inflammatory cells in the noise-exposed cochleae is still disputed. It has been proposed that inflammatory cells migrate into the cochlea to clear cellular debris resulting from noise-exposure and contribute to wound healing [46, 47]. However, this hypothesis is not supported by the fact that no inflammatory cells have been found in the organ of Corti, which is most severely injured by noise exposure. Loss of OHCs without any evidence of inflammatory response in the cochlea has also been documented [57]. The number of macrophages in the cochleae did not correlate with the damage level in the organ of Corti because an almost equal number of macrophages were found in cochleae exposed for 2 hours either to 112 or 120 dB SPL octave band noise (8–16 KHz) [46]. One possibility is that the inflammatory cells may activate a cochlear immune response and cause more cellular damage in the cochlea [47, 58]. A recent study demonstrated that the excessive infiltration of hematogenous macrophages caused more HC loss in the cochlea after kanamycin ototoxicity [59]. However, suppression of the inflammatory response in the cochlea could not prevent delayed HC loss although functional protection and increased ganglion neuron survival have been observed in a mouse AAT model [56]. Therefore, the inflammatory response may not directly contribute to the HC loss in the noise-damaged cochlea. Migration of inflammatory cells into the cochlea may initially be a response to the cellular damage in the organ of Corti, but these inflammatory cells cause cell death in the lateral wall [46]. This argument is supported by acute swelling and loss of intermediate cells in the stria vascularis and loss of type II and type IV fibrocytes in the spiral ligament after high levels (112 or 116 dB SPL for 2 hours) of noise exposure [60, 61]. Macrophages are also a source of NO production in early stages of wound healing [62] and may partially contribute to the formation of RNS in the cochlea. If this is the case, antioxidant treatment could reduce secondary injury to the cochlea induced by the inflammatory response. 

One question is why the majority of the CD45^+^ cells are located in the lateral wall of the cochlea, but only very few in the organ of Corti? The major sources of the inflammatory cytokines (i.e., IL-1*α*, IL-6, tumor necrosis factor *α*, macrophage inflammatory protein-2, and monocyte chemoattractant protein-1) are the fibrocytes and external sulcus cells located within the lateral wall [63, 64]. Expression of these inflammatory mediators is increased in the lateral wall in early stages after noise exposure [47, 55, 65], and these mediators would attract the inflammatory cells migrating into the lateral wall initially and then into other tissues of the cochlea.

### 4.2. The Role of RNS and ROS in the Cochlea after Noise Exposure and Antioxidant Treatment

In the present study, strong 4-HNE staining was found in IHCs and SCs 10 days after noise exposure; however, only weak positive staining was found in the OHC region. The positively stained SCs included inner and outer pillar cells, Deiters cells, and cells of Hensen and Boettcher (Figure 4(b)). This expression pattern was also found in the organ of Corti 21 days after noise exposure although the fluorescence intensity at 21 days was much weaker than that at 10 days (unpublished data). In a previous report, strong 4-HNE staining was detected in all cells of the organ of Corti in guinea pigs 10 days after 120 dB SPL, 5 hours of noise exposure [8]. The 4-HNE staining pattern in the present study is more like the staining pattern at day 7 after the noise exposure in that report (see Figure  5(d) in [8]). These results suggest that noise intensity might be an important factor affecting distribution of 4-HNE product in cells of the organ of Corti. Furthermore, 4-HNE was found in IHCs and SCs immediately after noise exposure [8] and still can be seen 21 days later, indicating that 4-HNE might form early and stay longer in IHCs and SCs than that in OHCs. Clearance of 4-HNE in the surviving OHCs begins around 14 days after the intense noise exposure [8] and probably before 10 days after a relative low dose of noise exposure used in the present study. Another possibility is that dying OHCs may be losing 4-HNE staining during the course of degeneration. It has been found that delayed degeneration of OHC develops for 4 weeks after noise exposure [66, 67].

However, how 4-HNE causes OHC loss is still unclear. Exposing organotypic cultures of the organ of Corti of 3 day old mouse pups to 4-HNE (75–150 uM), we found many more SCs undergoing necrotic and apoptotic cell death than OHCs, suggesting that 4-HNE may primarily cause SC death leading to OHC death (unpublished data). Majority of IHCs survived in the cochleae of chinchilla in both the noise exposure and noise/treatment groups (IHC loss was 1.90% and 1.06%, resp.) although IHC had strong 4-HNE staining, suggesting IHCs are less sensitive to 4-HNE damage. Significantly lower expression of 4-HNE was found in the organ of Corti of the noise/treatment group, suggesting that the antioxidant treatment inhibits the formation of 4-HNE in the organ of Corti. Consistent with our *in vivo* results, GSH treatment can protect inner ear HCs and hippocampal neurons from 4-HNE injury *in vitro* [68, 69]**. **Taken together, results in all of these reports and the present study indicate a relationship between 4-HNE formation and cochlear cell death in AAT [27] and that inhibition of 4-HNE formation in the cochlea may be one of the reasons why antioxidant treatment is effective in AAT. 

Strong NT staining was found in the spiral ligament of the noise exposure group, which was located in the cytoplasm of fibrocytes, the predominant cell type in this area. The spiral ligament is one of the nonsensory structures susceptible to AAT. Type II and IV fibrocytes in this area are significantly reduced after noise exposure [60, 70]**. **The strong expression of NT in the fibrocytes of the spiral ligament may be involved in the loss of type IV fibrocytes after noise exposure. Other free radicals (O^2−^, 8-isoprostane, NO, NT) were also detected in the spiral ligament immediately or few hours after noise exposure [1, 3, 5]. The function of fibrocytes in the spiral ligament was thought to be a purely passive role of structural support. However, increasing evidence suggests that they play more important and dynamic roles in the cochlea, such as potassium ion recycling, inflammatory reactions, and glutamate metabolism [71–73]. Loss of type IV fibrocytes may be a primary cause of age-related hearing loss and ultimate sensory cell degeneration in the C57BL/6J mouse [74]. After drug treatment, a significantly decreased expression of NT was found in the spiral ligament, suggesting that the drug treatment inhibits NT formation in the spiral ligament. The current results imply that antioxidant treatment may provide additional protection to the cochlea through inhibiting RNS formation in the spiral ligament. However, we did not find any significant NT staining in the organ of Corti or in the spiral ganglion 10 days after noise exposure, suggesting that formation of RNS may be not directly involved in OHC loss or neuron loss when examined at this time point. 

The results of 4-HNE and NT immunostaining showed no gradients for the various turns of the cochlea 10 days after noise exposure, yet the OHC loss and ABR threshold shifts were not uniform throughout the cochlea. These results suggest that noise exposure can promote free radical formation throughout the cochlea. However, the apical HCs are more resistant to free radicals than HCs in the basal and middle turns [75].

### 4.3. Possible Mechanisms of the Antioxidants in Treating AAT

In this three-drug combination, NAC provides cysteine for synthesis of GSH, works as a free radical scavenger, and inhibits cell death pathways [24, 28, 29]. ALCAR reduces ROS production and preserves mitochondria by serving as a precursor of acetyl-CoA, a mitochondrial energy substrate, and restoring a key mitochondrial lipid, cardiolipin, in oxidatively injured cells [16, 25, 29]. The nitrone, 4-OHPBN, is designed to scavenge free radicals as its phenyl ring reacts with hydroxyl radicals and may also decrease inflammation by inhibiting inflammatory mediators [32, 33, 76]. The precursor of 4-OHPBN, PBN, has been shown to have strong anti-inflammatory effects. PBN can decrease inducible cyclooxygenase (COX2) and iNOS mRNA levels, inhibit COX2 catalytic activity and lipopolysaccharide-mediated increase of nuclear factor KappaB (NF-KappaB) DNA binding activity [34]. COX2, iNOS, and NF-KappaB are important inflammatory mediators. Thus, 4-OHPBN is likely to be the major component in the three-drug combination to play an anti-inflammatory role. Therefore, the action of each of these three-drugs contributes separately to the attenuation of free radical formation and inflammatory responses in the cochlea to treat AAT. The potential mechanisms of therapeutic effects of each antioxidant will be assessed in our laboratory in an upcoming set of experiments.

## 5. Conclusion

The present study confirms the functional and HC protection of this three-antioxidant combination treatment in AAT. The results of our semiquantitative immunohistochemical analyses have demonstrated that the antioxidant treatment reduced not only formation of biomarkers for oxidative stress but also migration of mononuclear phagocytes into the cochlea. The finding in the present study that antioxidants can inhibit inflammatory responses in the cochlea suggests a new role for antioxidants in treating AAT in the future. These results have also confirmed that multiple damage mechanisms are involved in AAT and that simultaneous attenuation of these mechanisms at different sites in the cochlea using a combination of antioxidants to treat AAT may be effective [27].

## Abbreviations

ABR: Auditory brainstem response
ALCAR: Acetyl-L-carnitine
AAT: Acute acoustic trauma
COX2: Cyclooxygenase-2
DAPI: 4′,6-Diamidino-2-phenylindole
GSH: Glutathione
HC: Hair cell
4-HNE: 4-Hydroxy-2-nonenal
IHC: Inner hair cell
iNOS: Inducible nitric oxide synthase
NAC: *N*-Acetyl-L-cysteine
NT: Nitrotyrosine
NO: Nitric oxide
OHC: Outer hair cell
4-OHPBN: 4-hydroxy PBN
PBN: Phenyl *N*-*tert*-butylnitrone
PBS: Phosphate buffered saline
RNS: Reactive nitrogen species
ROS: Reactive oxygen species
SPL: Sound pressure level
SC: Supporting cell.
